# Screening of Common Retinal Diseases Using Six-Category Models Based on EfficientNet

**DOI:** 10.3389/fmed.2022.808402

**Published:** 2022-02-23

**Authors:** Shaojun Zhu, Bing Lu, Chenghu Wang, Maonian Wu, Bo Zheng, Qin Jiang, Ruili Wei, Qixin Cao, Weihua Yang

**Affiliations:** ^1^School of Information Engineering, Huzhou University, Huzhou, China; ^2^Zhejiang Province Key Laboratory of Smart Management and Application of Modern Agricultural Resources, Huzhou University, Huzhou, China; ^3^The Affiliated Eye Hospital of Nanjing Medical University, Nanjing, China; ^4^Department of Ophthalmology, Shanghai Changzheng Hospital, Huangpu, China; ^5^Huzhou Traditional Chinese Medicine Hospital Affiliated to Zhejiang University of Traditional Chinese Medicine, Huzhou, China

**Keywords:** fundus, retinal diseases, computer simulation, vision screening, optical imaging

## Abstract

**Purpose:**

A six-category model of common retinal diseases is proposed to help primary medical institutions in the preliminary screening of the five common retinal diseases.

**Methods:**

A total of 2,400 fundus images of normal and five common retinal diseases were provided by a cooperative hospital. Two six-category deep learning models of common retinal diseases based on the EfficientNet-B4 and ResNet50 models were trained. The results from the six-category models in this study and the results from a five-category model in our previous study based on ResNet50 were compared. A total of 1,315 fundus images were used to test the models, the clinical diagnosis results and the diagnosis results of the two six-category models were compared. The main evaluation indicators were sensitivity, specificity, F1-score, area under the curve (AUC), 95% confidence interval, kappa and accuracy, and the receiver operator characteristic curves of the two six-category models were compared in the study.

**Results:**

The diagnostic accuracy rate of EfficientNet-B4 model was 95.59%, the kappa value was 94.61%, and there was high diagnostic consistency. The AUC of the normal diagnosis and the five retinal diseases were all above 0.95. The sensitivity, specificity, and F1-score for the diagnosis of normal fundus images were 100, 99.9, and 99.83%, respectively. The specificity and F1-score for RVO diagnosis were 95.68, 98.61, and 93.09%, respectively. The sensitivity, specificity, and F1-score for high myopia diagnosis were 96.1, 99.6, and 97.37%, respectively. The sensitivity, specificity, and F1-score for glaucoma diagnosis were 97.62, 99.07, and 94.62%, respectively. The sensitivity, specificity, and F1-score for DR diagnosis were 90.76, 99.16, and 93.3%, respectively. The sensitivity, specificity, and F1-score for MD diagnosis were 92.27, 98.5, and 91.51%, respectively.

**Conclusion:**

The EfficientNet-B4 model was used to design a six-category model of common retinal diseases. It can be used to diagnose the normal fundus and five common retinal diseases based on fundus images. It can help primary doctors in the screening for common retinal diseases, and give suitable suggestions and recommendations. Timely referral can improve the efficiency of diagnosis of eye diseases in rural areas and avoid delaying treatment.

## Introduction

Common retinal diseases include retinal vein occlusion (RVO), high myopia, glaucoma, diabetic retinopathy (DR), and macular degeneration (MD) (1–5). DR and MD are high-incidence fundus diseases in China. According to statistics, patients with fundus diseases account for 54.7% of all blindness patients in China. There are more than three million people suffering from fundus diseases and more than two-thirds of patients with fundus diseases face blindness every year. Ophthalmologists use a non-mydriatic fundus color camera to obtain images of the fundus in these five common retinal diseases. A diagnosis is made by reading and interpreting the fundus images (6). At present, China's rural areas have inefficient transportation systems, poor medical conditions, and few professional ophthalmologists. Hence, patients with ophthalmopathy often only go to hospitals in the city to seek for treatment when the disease has already progressed; this may lead to delays in getting the best available treatment and may cause serious consequences for the patient.

A six-category model consisting of the normal retina and five common retinal diseases was designed to help patients with ophthalmopathy. This may be useful in rural areas for the preliminary diagnosis, accurate classification, and timely referral of retinal diseases.

In recent years, feature extraction methods using traditional machine learning have become a common method for diagnosing ophthalmologic diseases. The pertinent features of the ophthalmologic diseases were manually selected then identified through machine learning (7–13). Deep learning used convolutional neural networks to automatically extract image features; it obtained satisfactory results in the field of ophthalmology (14–23). Many researchers have used deep learning to diagnose retinal diseases using fundus images.

Nagasato et al. (24) compared the ability of machine learning technology and deep learning technology in the detection of branch RVO through the ultra-wide field-of-view fundus images; they found that deep learning technology had higher sensitivity and specificity. Li et al. (20) used convolutional neural networks to design a system based on macular images obtained through optical coherence tomography to identify the visual conditions of patients with high myopia; the said system had high area under the curve (AUC), sensitivity, and specificity. Ahn et al. (25) trained a new neural network model using fundus photos that can detect early and late glaucoma, with a high AUC. The Google team of Gulshan et al. (26) trained a deep learning model to diagnose DR through fundus images, and automatically graded DR; they obtained satisfactory results and carried out clinical trials upon patient follow-up. Yim et al. (21) combined a three-dimensional optical coherence tomography image and the corresponding automatic tissue map to design an artificial intelligence model to predict the progress of the other eye's conversion to exudative age-related macular degeneration of a patient with one eye diagnosed to have the said ophthalmologic disease. There were also a few studies that focused on the simultaneous screening of multiple diseases. Zheng et al. (15) used 2,000 fundus images to design a five-category model of common retinal diseases based on ResNet50; the model was able to diagnose common retinal diseases, except for macular degeneration (MD). Cen et al. (27) used deep neural networks to identify 39 retinal diseases and conditions that needed to be referred to higher facilities of care; although satisfactory results were achieved, the amount of data required for training was too large.

Our team in a previous study used the ResNet50 to create a five-category model that consisted of the normal fundus and four common fundus diseases (RVO, high myopia, glaucoma, and DR) (15), with an AUC above 0.92 and a kappa value of 89.33%. The retinal diseases in the previous study did not include MD because of its complicated features and different sub-types. However, since MD is a common retinal disease, our team included it in the new classification model used in this study.

This study designed a six-category model for common retinal diseases based on the EfficientNet model. It was used to detect the normal fundus and five common retinal diseases using fundus images. The model can help patients with ophthalmopathy in rural areas in their initial diagnosis of common retinal diseases for their prompt referral.

## Materials and Methods

### Data Source

The images used in this study were obtained from the Intelligent Ophthalmology Database of the Ophthalmology Hospital of Nanjing Medical University. These images were obtained by various types of non-mydriatic fundus cameras. This study used the EfficientNet model to train a six-category model for common fundus diseases. A total of 2,400 fundus images were used as training data; there were 400 fundus images for each retinal disease and 400 images of normal fundus. A total of 1,315 fundus images were used as test data. The research had no restrictions on the sex and age of the patients who had their fundus images taken. The relevant personal information of the patients were removed before the fundus images were delivered to the researchers. Therefore, this research did not determine the demographic information of the patients who had their fundus images taken.

**Figure 1:**
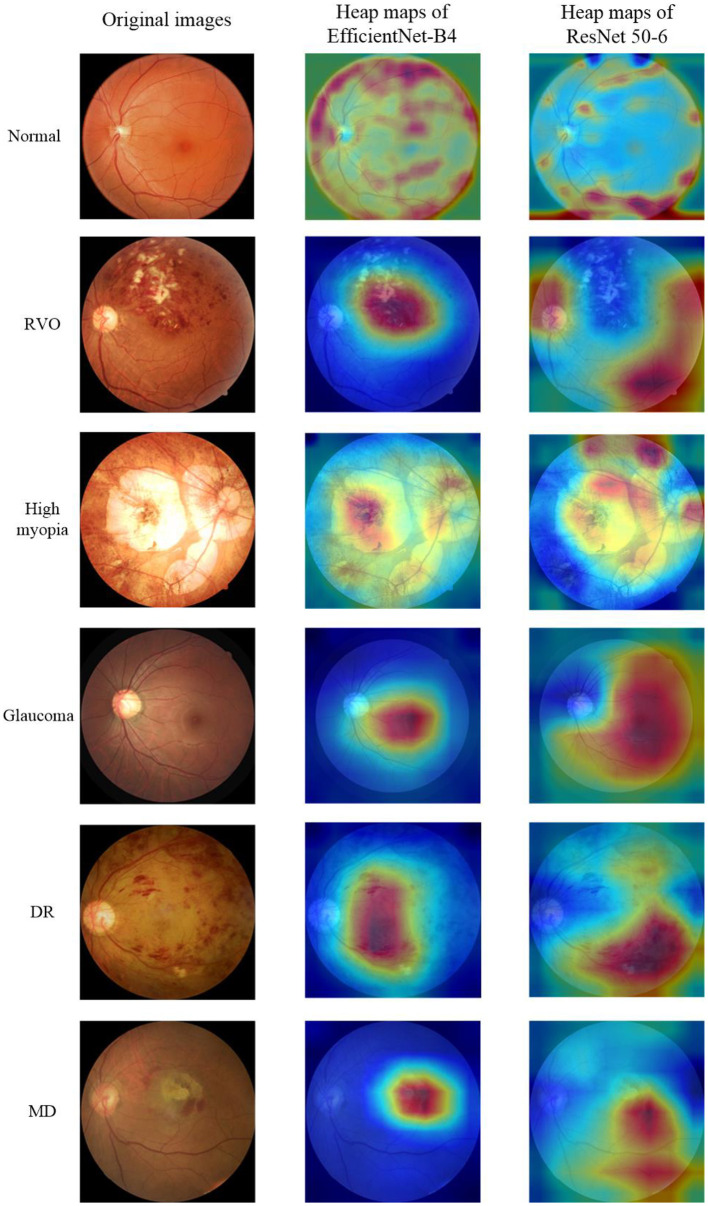
Original Images and heap maps of the normal fundus and five common retinal diseases.

The fundus images provided by the cooperative hospital were of high quality. The actual diagnoses of the images were given at the same time and were regarded as the diagnoses from the expert ophthalmologist. Two other experienced ophthalmologists independently diagnosed the fundus images. If the two ophthalmologists had the same diagnosis, then it was regarded as the final diagnosis. However, if the two ophthalmologists had different diagnoses, then the expert ophthalmologist would assess the fundus image and gave the final diagnosis. The fundus images only had one disease and did not contain multiple retinal diseases. A fundus image could only be assessed as normal or diagnosed with one of the five common retinal diseases (RVO, high myopia, glaucoma, DR, and MD). The normal fundus image and the fundus images of the five common retinal diseases are shown in the first column of Figure 1.

### Model Training

**Figure 2:**
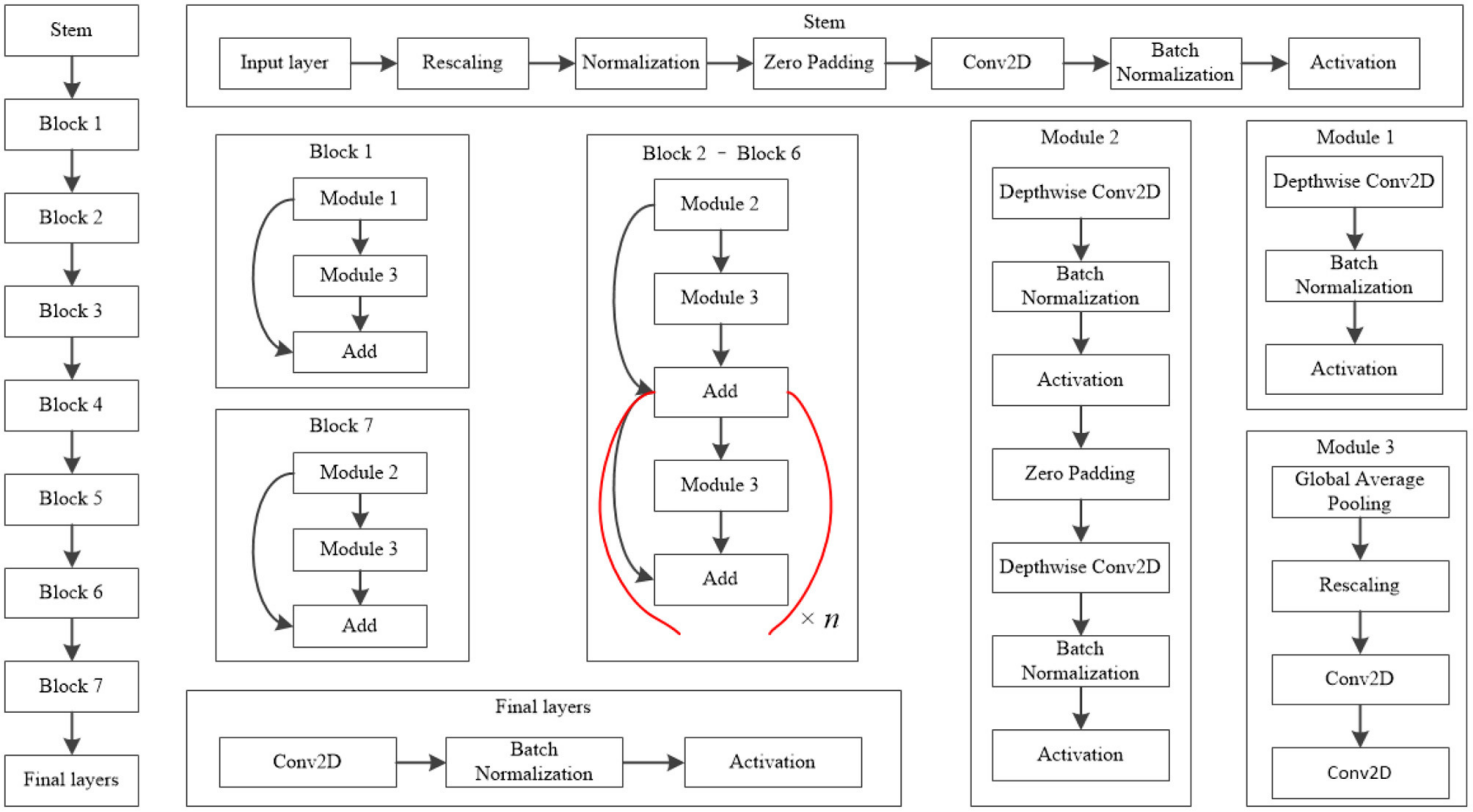
Model structure of EfficientNet-B4. The values of *n* in this figure of Block 2–Block 6 is 2, 2, 4, 4, and 6, respectively.

**Figure 3:**
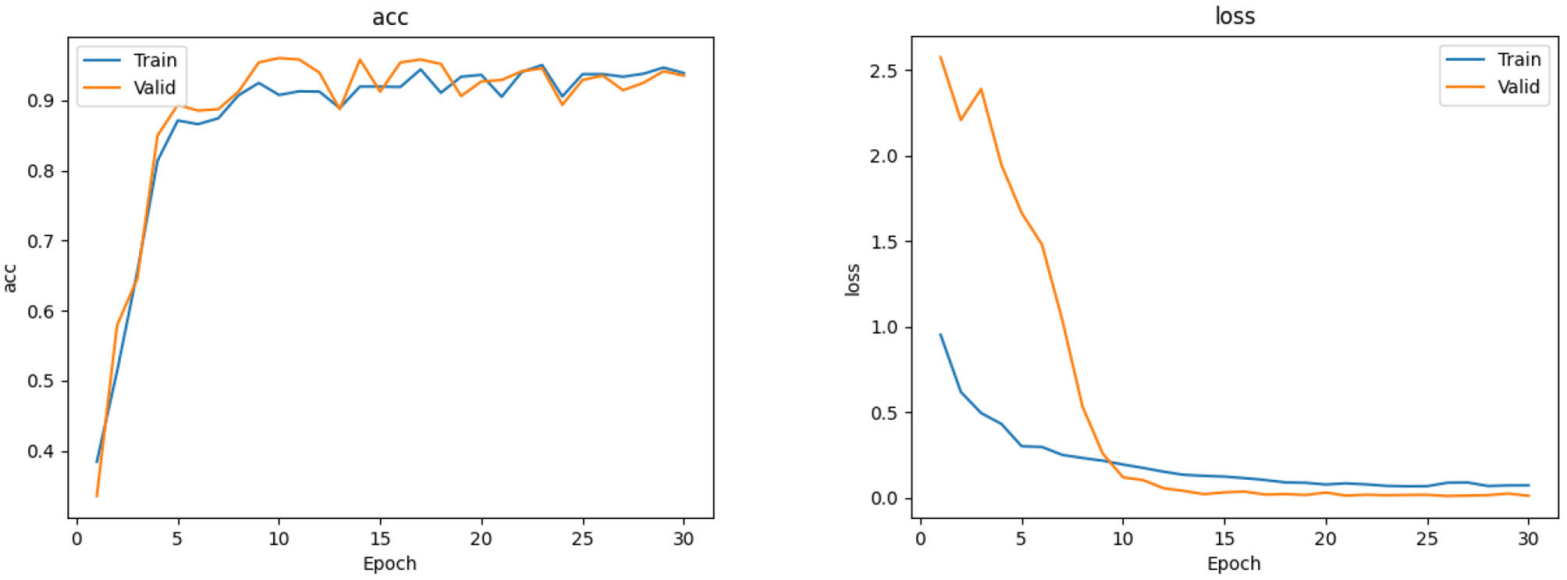
The accuracy and loss curves of EfficientNet-B4.

The EfficientNet-B4 model (28) was used to classify the normal fundus and the five common retinal diseases using the fundus images. The EfficientNet model was proposed by Google. EfficientNet-B0 provided the basebone; its depth, width, and resolution were jointly adjusted to obtain the other models. Finally, eight models with different parameters, from EfficientNet-B0 to EfficientNet-B7, were created. EfficientNet-B4 is mainly composed of one stem, seven blocks, and one final layer. The seven blocks mainly included modules 1, 2, and 3. All modules were mainly composed of the convolutional layer, pooling layer, and activation layer. The model structure and learning curves of EfficientNet-B4 is shown in Figures 2, 3, respectively.

The classic classification model of deep learning also included other models like VGG16 (28) and ResNet50 (29), among others. Their basic network structure mainly included convolutional layers, pooling layers, and fully connected layers. Our team previously used ResNet50 to train a five-category intelligent auxiliary diagnosis model for common retinal diseases (normal fundus and four common retinal diseases, excluding MD in that study) (15). Hence, in this study, the ResNet50 model was used to classify the normal fundus and the five common retinal diseases. The results of the ResNet50 model were compared with the results of the EfficientNet-B4 model.

The six-category model of common retinal diseases only changed the output layer category; there were no changes on the original network structure of the EfficientNet-B4 model during training. The initial parameters of the six-category model obtained after training were transferred to the ImageNet Large Scale Visual Recognition Challenge (30) to improve the initial performance of the model. Then, 2,400 fundus images were used to train the model iteratively to obtain the best weighted parameters. Finally, the six-category model of common retinal diseases was obtained.

### Statistical Analysis

SPSS version 22.0 statistical software was used for statistical analysis. The receiver operating characteristic curve was used to analyze the diagnostic performance of the model, and kappa value was used to test the consistency of the diagnosis between the expert and the model. A kappa value of 0.61–0.80 indicated significant consistency, and >0.80 indicated high consistency. The sensitivity, specificity, F1-score, 95% confidence interval, AUC and other indicators of the six-category model of the normal fundus and the five common retinal diseases were calculated. The classification effect of the AUC values were interpreted as follows: >0.85, high; 0.7–0.85, average; and 0.5–0.7, poor.

## Results

There were 1,315 fundus images used to test the six-category models of the common retinal diseases. The expert ophthalmologist diagnosed 300 fundus images as normal, 162 fundus images as RVO, 308 fundus images as high myopia, 126 fundus images as glaucoma, 238 fundus images as DR, and 181 fundus images as MD. The EfficientNet-B4 six-category model diagnosed 301 fundus images as normal, 171 fundus images as RVO, 300 fundus images as high myopia, 134 fundus images as glaucoma, 225 fundus images as DR, and 167 fundus images as MD. The ResNet50 six-category model diagnosed 301 fundus images as normal, 168 fundus images as RVO, 265 fundus images as high myopia, 161 fundus images as glaucoma, 221 fundus images as DR, and 199 fundus images as MD. The results of the EfficientNet-B4 model and the ResNet50 model are shown in Tables 1, 2, respectively.

**Table 1:** Diagnostic results of the EfficientNet-B4 model.

**Clinical**	**EfficientNet-B4 Model**						
	**Normal**	**RVO**	**High myopia**	**Glaucoma**	**DR**	**MD**	**Total**
Normal	300	0	0	0	0	0	300
RVO	0	155	0	1	4	2	162
High myopia	0	1	296	7	2	2	308
Glaucoma	0	0	1	123	0	2	126
DR	1	9	1	0	216	11	238
MD	0	6	2	3	3	167	181
Total	301	171	300	134	225	184	1,315

*RVO, retinal vein occlusion; DR, diabetic retinopathy; MD, macular degeneration*.

**Table 2:** Diagnostic results of the ResNet50 model.

**Clinical**	**ResNet50 Model**						
	**Normal**	**RVO**	**High myopia**	**Glaucoma**	**DR**	**MD**	**Total**
Normal	299	0	0	0	0	1	300
RVO	0	133	1	3	15	10	162
High myopia	0	2	259	18	1	28	308
Glaucoma	0	1	0	108	3	14	126
DR	0	15	0	9	191	23	238
MD	2	17	5	23	11	123	181
Total	301	168	265	161	221	199	1,315

*RVO, retinal vein occlusion; DR, diabetic retinopathy; MD, macular degeneration*.

Compared with the expert diagnosis group, the EfficientNet-B4 six-category model had 95% sensitivity for the diagnoses of RVO, high myopia, and glaucoma, while 90% sensitivity was found for the diagnoses of DR and MD. The specificity for diagnosing the five retinal diseases was approximately 99%. All the AUCs were above 95%, and the kappa value was 94.61%; this implies a high consistency of the model. The ResNet50 six-category model (ResNet 50-6) had >80% sensitivity for the diagnoses of RVO, high myopia, glaucoma, and DR. However, the sensitivity of the model for diagnosing MD was only at 67.96%. There was >93% specificity for diagnosing the five retinal diseases. All the AUCs were above 80%, and the kappa value was 81.31%; thus, there was high consistency of the model. The ResNet50 five-category model (15) (ResNet50-5) was made by our team; it is a five-category intelligent auxiliary diagnosis model of common retinal diseases. All the indicators for diagnosing the normal fundus images of the three models can reach 99%. The evaluation index results of the three models are shown in Table 3.

**Table 3:** Evaluation of the index results of the three models.

**Model**	**Evaluation indicators**	**Normal**	**RVO**	**High myopia**	**Glaucoma**	**DR**	**MD**
EfficientNet-B4	Sensitivity	100%	95.68%	96.10%	97.62%	90.76%	92.27%
	Specificity	99.90%	98.61%	99.60%	99.07%	99.16%	98.50%
	F1-score	99.83%	93.09%	97.37%	94.62%	93.30%	91.51%
	AUC	1	0.971	0.979	0.983	0.950	0.954
	95% CI	0.998–1	0.953–0.990	0.966–0.991	0.968–0.999	0.928–0.971	0.931–0.977
	Kappa	94.61%					
	Accuracy	95.59%					
ResNet50-6	Sensitivity	99.67%	82.10%	84.09%	85.71%	80.25%	67.96%
	Specificity	99.80%	96.96%	99.40%	95.54%	97.21%	93.30%
	F1-score	99.50%	80.61%	90.40%	75.26%	83.22%	64.74%
	AUC	0.997	0.895	0.917	0.906	0.887	0.806
	95% CI	0.993–1	0.860–0.931	0.893–0.942	0.870–0.943	0.857–0.918	0.765–0.848
	Kappa	81.31%					
	Accuracy	84.64%					
ResNet50-5 (15)	Sensitivity	99.33%	87.65%	87.34%	95.24%	88.24%	—
	Specificity	100.00%	96.50%	99.52%	96.43%	97.66%	—
	F1-score	99.67%	84.02%	92.60%	85.11%	89.55%	—
	AUC	0.996	0.921	0.934	0.958	0.929	—
	95% CI	0.991–1	0.890–0.951	0.912–0.956	0.936–0.981	0.905–0.954	—
	Kappa	89.33%					
	Accuracy	90.59%					

*AUC, area under the curve; CI, confidence interval; RVO, retinal vein occlusion; DR, diabetic retinopathy; MD, macular degeneration*.

**Figure 4:**
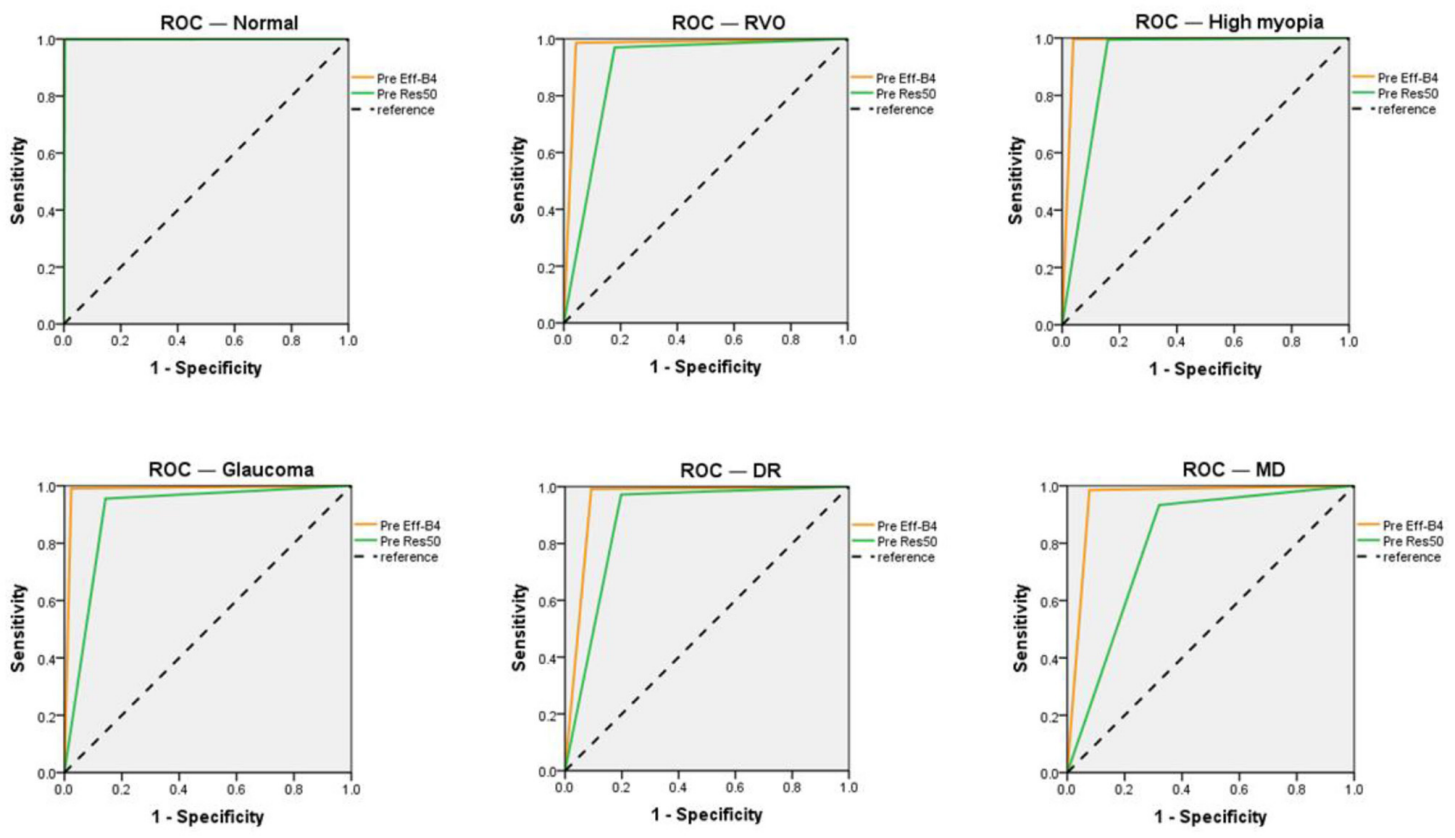
ROC of the EfficientNet-B4 and ResNet 50-6 for normal fundus and the five common retinal diseases.

The EfficientNet-B4 and ResNet50-6 models are six-category models, while the ResNet50-5 model is a five-category model for common retinal diseases. The EfficientNet-B4 model was found to be superior to the ResNet50-6 and ResNet50-5 models in terms of sensitivity and specificity in the diagnoses of RVO, high myopia, glaucoma, and DR. The ResNet50-5 model could diagnose more accurately RVO, high myopia, glaucoma, and DR than the ResNet50-6 model. Figure 3 shows the accuracy and loss curves of Efficient-B4. Figure 4 shows the comparison of ROC curves between the EfficientNet-B4 model and the ResNet50-6 model for the assessment of the images of the normal fundus and of the five common retinal diseases.

This study used Grad-CAM (31) to make heat maps for the EfficientNet-B4 and ResNet 50-6 models, as shown in the second and third columns of Figure 1, respectively. It can be seen from the figure that the focus area marked by the heat map of the EfficientNet-B4 model is more accurate, while the accuracy of the focus area marked by the ResNet 50-6 model is slightly worse. In this study, when the same algorithm is used to obtain the heat map, the better the evaluation index of the model, the more accurate the heat map area obtained.

## Discussion

In 1998, LeCun et al. (32) proposed the LeNet-5 model that used convolutional neural networks to recognize handwritten digits. Their study laid the foundation for the basic convolutional neural network (CNN) architecture of convolution, pooling, and fully connected layers. After the year 2012, deep learning had developed rapidly. The AlexNet (33) model, VGG model, GoogleNet (34) model, and ResNet model had obtained the best results for the image classification or object detection using the ImageNet Large Scale Visual Recognition Challenge. In 2019, Google researchers proposed the EfficientNet model. First, the MnasNet (35) method was used to design EfficientNet-B0 that served as the basebone of EfficientNet-B1 to B7. The network depth, width, and resolution were refined in the succeeding versions. In this study, the EfficientNet model was selected to classify the normal and the five common retinal diseases based on the fundus images. Compared with other models, EfficientNet showed a better ability to extract the internal features, hence the classification and diagnoses were improved.

Tables 1, 2 shows that the EfficientNet-B4 model had a better diagnostic ability as compared with the ResNet50-6 model. The main reason could be explained by the complexity of the fundus images; there were inconsistent focus areas and varying characteristics of the different retinal diseases. The ResNet 50 model had 50 layers, while the EfficientNet-B4 model had deeper layers. Thus, the deep features of the fundus image could be extracted through operations, such as convolution. These deep features could help increase the accuracy of the model's assessment of the fundus image's diagnosis. However, the models used in this study had poor diagnostic results for MD. It was misdiagnosed as other diseases in large numbers because of its complicated manifestation in fundus images. The models had a difficulty in determining MD from other types of macular lesions, hence leading to misdiagnosis.

Our team had proposed a five-category model for normal fundus images and the four common retinal diseases based on the ResNet50 model. MD was not included in the four common retinal diseases in that study. Moreover, the results of the related retinal diseases were compared with the results of the five-category model. The results of the ResNet50-5 model in Table 3 are based on the five-category model. The addition of new categories will increase the difficulty of the model's identification of the target. The accuracy of the classification of the results by the model would decrease. This would apply to the features of other retinal diseases that may contain the features of MD that would make the diagnosis of MD more difficult. Consequently, this increases the probability of misdiagnosing MD as other types of retinal disease.

Some researchers had also done research on multi-class fundus diseases. Karthikeyan et al. (36) used deep learning to detect 12 major retinal complications, and the verification accuracy was 92.99%. Wang et al. (37) used the EfficientNet model to do multi-label classification research and the accuracy was 92%. The accuracy of the EfficientNet-B4 model in this study is 95.59%, but the images only had single labels, which was normal and five common fundus diseases. Other fundus diseases were usually classified into five fundus diseases, and then they would be diagnosed again by a doctor.

Tables 1–3, show that the two six-category models could diagnose normal fundus images. It is rare for normal fundus images to be diagnosed with retinal disease. There was one or two images with retinal disease that were diagnosed as normal images for the two models; the misdiagnosis mainly occurred in the fundus images of DR and MD. In the typical process of making a diagnosis, the doctor would need to confirm the results after the preliminary diagnosis using the classification model. The DR and MD lesions were relatively apparent, and even non-ophthalmologists could make good judgments after basic training. Therefore, the missed diagnosis of these two retinal diseases will be greatly reduced after the doctor's confirmation

The six-category model for common fundus diseases based on EfficientNet-B4 had high sensitivity and specificity for diagnosing normal fundus and five common retinal diseases. Therefore, it may be suitable for the primary diagnosis of common retinal diseases at the primary hospital. It may help increase diagnostic accuracy in primary care and support early detection, diagnosis, treatment, and referral. However, the model also had some shortcomings. For example, the sensitivity of diagnosing DR and MD was lower than in the other retinal diseases. The model could be further improved by increasing the number of training images to attain a better diagnostic performance.

## Data Availability Statement

The raw data supporting the conclusions of this article will be available from the corresponding author on reasonable request.

## Author Contributions

SZ and BZ wrote the manuscript. CW, MW, and WY reviewed the manuscript. BL and QJ trained the model. RW and QC collected and labeled the data. All authors issued final approval for the version to be submitted.

## Funding

This research supported by the National Natural Science Foundation of China (No. 61906066), Natural Science Foundation of Zhejiang Province (No. LQ18F020002), Science and Technology Planning Project of Huzhou Municipality (No. 2016YZ02), and Nanjing Enterprise Expert Team Project. The Medical Science and Technology Development Project Fund of Nanjing (Grant No. YKK21262); Teaching Reform Research Project of Zhejiang Province (jg20190446).

## Conflict of Interest

The authors declare that the research was conducted in the absence of any commercial or financial relationships that could be construed as a potential conflict of interest.

## Publisher's Note

All claims expressed in this article are solely those of the authors and do not necessarily represent those of their affiliated organizations, or those of the publisher, the editors and the reviewers. Any product that may be evaluated in this article, or claim that may be made by its manufacturer, is not guaranteed or endorsed by the publisher.
